# Supplementary material for: Patterns of Intron Gain and Loss in Fungi
Source: PLoS Biol. 2004 Nov 30;2(12):e422. doi: 10.1371/journal.pbio.0020422 (PMC532390; doi:10.1371/journal.pbio.0020422)
Supplement: Table S1 — Also available at http://genes.mit.edu/NielsenEtAl/. (4.3 MB ZIP). [file pbio.0020422.st001.zip › NielsenEtAl/html/173.html]

AN5880.1.NCU04387.1.MG01556.1.FG09587.1


```
 CLUSTAL W (1.82) Multiple Sequence Alignments - Introns Inserted


Sequence 1: NCU04387.1	124 aa
Sequence 2: MG01556.1	126 aa
Sequence 3: FG09587.1	123 aa
Sequence 4: AN5880.1	124 aa
Alignment Length: 126 aa
Number Identitical Residues: 88 aa
Alignment Score (without introns) 3708


MG01556.1 	MSNNAMAADSK~DRQFLAVIGDE0D~SVTGLLLAGIG0HVTPAPDSQKNFLVVDNKTDTA
NCU04387.1	MAT--SQADAR~DRQFLAVIGDE0D~SVTGLLLAGIG0HVTAPPDSQKNFLVVDNKTDNA
FG09587.1 	MAS---QADYK~DRQFLAVIGDE0D~SVTGLLLAGIG0HVTTGADAQKNFLVVDGKTDTS
AN5880.1  	MAAG--AVAYK0DRQFLAVIGDE~D2SVTGLLLAGIG0HVTGPPDSQRNFLVVDAKTENS
          	*: .   .  : *********** * *********** ***  .*:*:****** **:.:

MG01556.1 	AIESAFQKFTTERKDIGIVLINQH0IADRIRHIVDTYTAAFPAVLEIPSKDHPYDPEKDS
NCU04387.1	AIEAAFDRFTTERKDIGIVLINQH0IADRIRHRVDTHTAAFPTVLEIPSKDHPYDPEKDS
FG09587.1 	AIEAAFDRFTEDRKDIGIVLINQH0IAERIRHRIDTYTAAFPAVLEIPSKDHPYDPEKDS
AN5880.1  	TIENAFQNFTQERNDIAVLLINQH0IAERIRHVIDSYSEAFPAVLEIPSKDHPYDPEKDS
          	:** **:.** :*:**.::***** **:**** :*::: ***:*****************

MG01556.1 	VLRRVRRLFGE
NCU04387.1	VLRRVRRLFGE
FG09587.1 	VLRRVRRLFGE
AN5880.1  	VLKRVRRLFGE
          	**:********
```
